# A glimmer of hope for a neglected disease - How Chagas disease associative movement is driving progress towards disease control and elimination

**DOI:** 10.1590/0074-02760220083chgsb

**Published:** 2022-10-24

**Authors:** Carolina Batista

**Affiliations:** 1 Head of Global Health Affairs, Baraka Impact Finance. Former International Board Member, Médecins Sans Frontières (MSF)

“The person who feels the pain is the one who has to fight”, or as originally said in Portuguese “Quem sente a dor é quem tem que lutar”. I can never forget Mrs. Amelia dos Santos’ words when I first met her. It was October 2010, in the Olinda city, Northeast of Brazil, not far from where the first Chagas disease (CD) patient association had been created back in 1987. The occasion was the first Federation of Associations of People Affected by Chagas Disease (FINDECHAGAS) international assembly, as referred to in the article by Wilson Alves de Oliveira Junior et al.

As I was still trying to process the power and relevance of her words, I looked around and saw many faces that had been directly or indirectly affected by CD. For them it had never been the silent disease we hear and read about. Their lives had been touched, their health compromised and a mix of feelings, ranging from fear, frustration to anger or sorrow inevitably emerged after the diagnosis. Mrs. Cristina, a Quechua indigenous from Bolivian, also shared her story with the assembly participants. I had previously met her in the remote mountains of Aiquile, Bolivia, at one of Médecins Sans Frontières (MSF) projects I worked at.^1^ Her passion and engagement always inspired me. Her family had been affected by CD throughout generations. As far back in her ancestors’ line as she was able to remember. She could always tell about a family member who had suffered from CD. Stories such as “my grandfather went to harvest potatoes, felt a sharp pain in his chest and suddenly died” were recurrent. And not only from Cristina’s family, but according to her, from the other villagers as well. 

This was the first FINDECHAGAS meeting I joined, the first of many that I subsequently had the honour to engage and learn. Even if the countries where meetings occurred changed along the years, the demands from the affected people were always similar; they all wished to talk about their experiences and to have their voices heard. These real “Chagas experts”, as I like to call them, could very eloquently teach academics and other public health specialists about their journey through the conundrum of health systems in their home societies, which were not prepared to properly diagnose and provide care for this neglected disease. 

The article “How people affected by Chagas disease have struggled with their negligence: history, associative movement and World Chagas Disease Day” provides a comprehensive description of how individuals have organised themselves over the years despite the challenges and lack of representation. Currently there are between six and seven million people estimated to be infected with *Trypanosoma cruzi.* ^2^ These numbers fail to show the faces, living conditions, life experiences and invisibility that have kept individuals as figures with no first and last name for so long. The diversity of faces, accents and addresses of individuals that are part of FINDECHAGAS demonstrate the globalisation of the disease and how migration has defined the need for new public health policies and integration of affected individuals in health programs to successfully address their needs. Successful experiences in Spain, Switzerland and the United States, for instance, have demonstrated that putting the individual in the centre of health response, to ensure the right to care, regardless of their migration status, should be prioritised in the public health agenda.^3^ ^-^ ^5^ It should also serve as a means to mitigate xenophobia and discourses about migrants as those bringing diseases across borders, what has been proved to be false several times. Patient and affected individuals associations are of paramount importance to ensure their representation and inclusion in migration debates and policymaking initiatives that aim at addressing exclusion and prejudice against those who had to leave their countries in search of better lives.

In 2016, I was closely involved with the creation of the Brazilian Social Forum to Combat Infectious and Neglected Diseases. This initiative brings together different movements of the organised civil society focused on ensuring fundamental rights and in the construction of public policies and research for neglected and infectious diseases throughout the country.^6^ The forum was built on the experience of those affected by infectious and neglected diseases, such as CD, leishmaniasis, schistosomiasis, leprosy, hepatitis and their journey to access diagnosis, proper care and socioeconomic support to deal with such conditions. The newly created forum also enabled broader participation and inclusion of individuals affected by conditions such as leishmaniasis and others that had not yet a formal organisation due to lack of support and social capital. It did not take long to realise that many of the struggles and challenges experienced by individuals and families were similar, regardless of the pathogen they had been affected by. After all, these were for the most part, “neglected individuals”, or as Hotez^7^ describes the vicious cycle of “forgotten people, forgotten diseases”. This reiterated the common features and determinants of these conditions and how the social fabric in which these individuals live determine their access, or lack of it, to proper healthcare, social support and opportunities to thrive in life. It was evident that this was an unprecedented opportunity to foster dialogue, share experiences and learn lessons from the various groups and their journeys.

We have all witnessed how coronavirus disease 2019 (COVID-19) has disproportionately affected vulnerable individuals across the globe. The reasons for this are multidimensional; nevertheless, one important reason is the historic mistrust of the biomedical community, which is a consequence of long-standing exclusion by healthcare systems, lack of access to care, and neglect.^8^ Similarly, individuals affected by CD have been historically excluded and disengaged in designing programs to address their needs. From the letters sent by CD affected people to Dr Dias many decades ago, one can see the urge to access care and lack of proper messaging around the disease. Looking forward, the advent of digital health and social media that have been quickly adopted in recent years start to be part of the arsenal for solutions to address CD, including co-designed information and education messages to fight misinformation and stigma that still persists. 

The recent progress achieved for CD, including the establishment of the World Chagas Disease Day, on April 14th, the consolidation of FINDECHAGAS, initiatives to eliminate congenital CD and break the epidemiological silence around the disease, bring a glimmer of hope for people like Mrs. Amelia, Mrs. Cristina, Mr. Manoel and many others I have met along the years. The inspiring achievements led by associations should also serve as a reminder that collective action, citizen agency, collaboration, persistence and even resilience are key elements to achieve long-lasting impact and will remain key to breaking the cycle of neglect and silence around CD. 

Comments on the article: de Oliveira Jr WA, Gómez i Prat J, Albajar-Viñas P, Carrazzone C, Kropf SP, Dehousse A, et al. How people affected by Chagas disease have struggled with their negligence: history, associative movement and World Chagas Disease Day. Mem Inst Oswaldo Cruz. 2022; 117: e220066.
